# New onset episodic vertigo as a presentation of vestibular neuritis

**DOI:** 10.3389/fneur.2022.984865

**Published:** 2022-10-12

**Authors:** Lu Tang, Weiwei Jiang, Xiaoshan Wang

**Affiliations:** Department of Neurology, The Affiliated Brain Hospital of Nanjing Medical University, Nanjing Medical University, Nanjing, China

**Keywords:** peripheral vestibular vertigo, vestibular neuritis, video head impulse test (vHIT), new onset episodic vertigo, overt saccades

## Abstract

**Objective:**

Vestibular neuritis (VN) is a common peripheral cause of acute vestibular syndrome, characterized by sustained vertigo and gait instability, persisting from 1 day to several weeks. With the widespread use of comprehensive vestibular function tests, patients with VN and non-sustained vertigo have drawn attention. In this study, we retrospectively analyzed the clinical presentation of patients with VN and episodic vertigo, aiming to expand the atypical clinical features of VN.

**Methods:**

This retrospective study enrolled 58 patients with VN. Among them, 11 patients with more than 3 remissions per day, each lasting over 1 h were assigned to the episodic vertigo (EV) group, and 47 subjects without significant relief into the sustained vertigo (SV) group. Demographic information, clinical manifestations and data of supplementary examinations were collected and statistically analyzed. These patients were followed up 1 year after discharge to gather prognostic information.

**Results:**

The incidence of spontaneous nystagmus (SN) and proportion of severe vertigo (Dizziness Handicap Inventory questionnaire score >60) in the SV group were significantly higher than those in the EV group. Spearman correlation showed that with a longer disease course, the velocity of overt saccade was smaller (*p* < 0.05, Rs = −0.263) in all patients with VN.

**Conclusion:**

The non-sustained manifestations in VN overlap with a wider spectrum of other vestibular disorders and stroke-related vertigo, which add an additional layer of complexity to the differential diagnosis of new onset episodic vertigo. By retrospectively analyzing the clinical characteristics and vHIT parameters, our study has expounded on the atypical features and potential pathophysiological mechanism of episodic syndromes in VN. VOR gain and saccades measured by vHIT could be reliable indicators for vestibular rehabilitation process.

## Introduction

Vestibular neuritis (VN) is characterized by the sudden onset of sustained vertigo and gait instability. These symptoms develop acutely in minutes or hours. VN affects males and females equally, with a peak onset age of 40–50 years (1, 2). It is always accompanied by nausea or vomiting, head motion intolerance, and nystagmus. The sense of imbalance and unsteadiness may linger for weeks (1, 3). As the second common peripheral cause of acute vestibular syndrome next to benign episodic positioning vertigo, VN is diagnosed in 3.2–9% of patients visiting clinics because of dizziness (3). Inflammatory etiology of VN has long been hypothesized on the basis of its association with respiratory tract infections and its frequent occurrence in epidemics (3, 4). Inflammatory activation after infections leads to a systemic reaction reducing microvascular perfusion and vestibular organ infarction, thus causing the loss of vestibular function (5). Possible comorbidity with herpes simplex virus type 1 reactivation or influenza virus infection has also been proposed (6, 7). Nevertheless, evidence of systemic viral infection based on seroconversion remain unconvincing (3).

As defined by the Committee for the Classification of Vestibular Disorders of the Bárány Society, patients are suspected of acute unilateral vestibulopathy / VN when they have prolonged vertigo with unsteadiness, nausea/vomiting and/or oscillopsia for days or weeks, and spontaneous horizontal–torsional nystagmus with the quick phase beating away from the lesion side (8, 9). The diagnosis of VN is generally based on the comprehensive interpretation of clinical symptomatology, laboratory evaluation and reasonable exclusion of other disorders, such as acute central lesions or peripheral audiological vestibular disorders (9, 10). Neurological signs are very important for the diagnosis of VN. Performed by trained specialists, the HINTS exam (Head Impulse, Nystagmus and Test of Skew) is a series of three bedside ocular motor tests that can be used to differentiate central and peripheral symptoms in patients with acute vestibular syndrome (11–14). Head impulse tests (HITs) could identify the function of the six semicircular canals over a high frequency range, which is similar to those of head movements (15–17). With a video-monitoring system, the video HITs (vHITs) provide objective measurement of the vestibular- ocular- reflex (VOR) gains and saccade parameters by capturing eye and head movements (18). Spontaneous nystagmus (SN) suppressed by fixation is an important clinical sign in patients with VN, which is caused by an imbalance average firing rate in the vestibular nerve on both sides (19). Skew deviation is a sign of an abnormal otolith-ocular reflex (OOR). Large amplitude skew deviation and the ocular tilt reaction (OTR) are commonly seen in central lesions (14, 20, 21), but rarely in VN (22).

Vestibular function evaluation is also useful for the diagnosis of VN. For example, caloric tests are used in investigating the function of the horizontal semicircular canal in the low frequency range (~0.003 Hz), in which a canal paresis (CP) of ≥ 25% is the diagnostic hallmark of VN (10, 23). Other quantitative assessments, such as vestibular evoked myogenic potentials (VEMPs) (24, 25), subjective visual vertical (SVV) (26) and vestibular autorotation test (VAT) (27), are also helpful tools for evaluating the diagnosis and prognosis of VN. Although there is no definite examination for VN, these approaches can help evaluate different portions of the peripheral vestibular system and appear to complement each other.

With the widespread application of neurological examination and vestibular electrophysiology tests, we have previously observed the patients diagnosed with VN experienced episodic vertigo. A few studies have revealed the atypical features in VN, such as transient dizziness or prodromal unsteadiness before severe prolonged vertigo, overturning the general awareness of VN symptoms (3, 28, 29). Reportedly, VN is the third common trigger after benign paroxysmal positional vertigo (BPPV) and vestibular migraine of secondary functional dizziness (9). Nevertheless, appropriate rehabilitation promoted in time could benefit patients with less sequelae and functional disturbance (30, 31). Since transient ischemic attack (TIA) of vertebrobasilar territory could also manifest as recurrent vertigo, we sought to retrospectively analyze the clinical features of VN patients with episodic vertigo, aiming to alert awareness of the diagnostic confusion.

## Methods

### Participants

**Figure 1:**
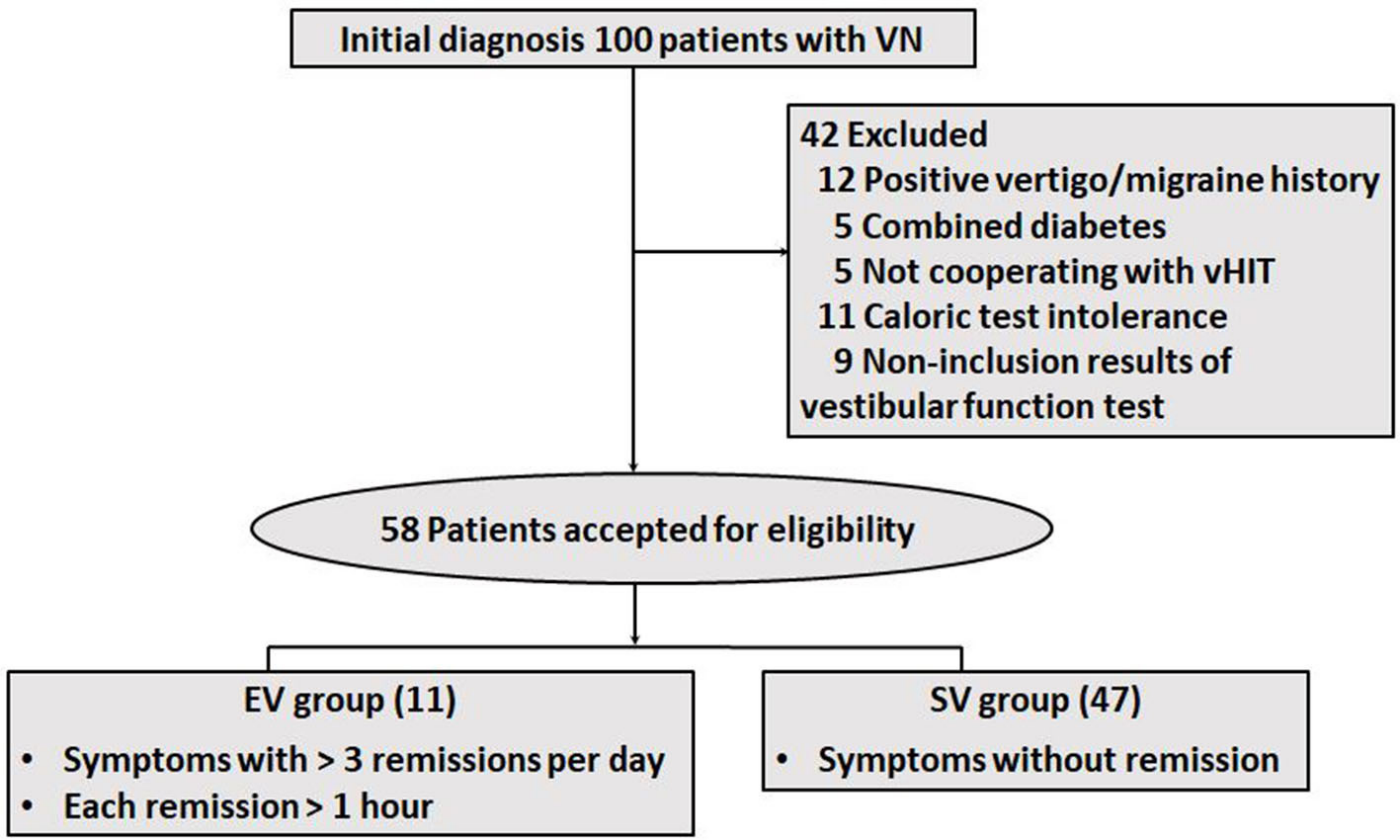
Flow chart for enrolling and grouping patients.

A total of 100 patients diagnosed with VN were screened for inclusion from January 2018 to June 2022. They were hospitalized in the Neurology Division Vertigo Center at the Affiliated Brain Hospital of Nanjing Medical University. All subjects were inquired about their symptoms and completed the Chinese version of the original dizziness handicap inventory (DHI) questionnaire at initial visit. We emphasized the symptomatic persistence at rest when taking the semeiology of a case. Patients who met all of the following criteria were enrolled in this study: (1) patients without vertigo history by complaint of a sudden onset of vertigo (more than 24 h); (2) patients with positive first degree vestibular nystagmus and negative skew deviation test; (3) patients without brain lesions according to magnetic resonance imaging (MRI) and diffusion-weighted imaging; (4) patients with either horizontal or vertical vHITs gain value of <0.8 and corrective saccades peak velocity of >100°/s (32– 34); (5) patients with affected horizontal semicircular canals, vHITs showing corrective saccades on the side of slow-phase SN, and CP of >25% according to caloric examination. The following patients were excluded: (1) patients with medical history of diabetes, migraine, vertigo or anxiety; (2) patients with auditory symptoms, such as hearing loss, tinnitus, or ear fullness on either side; and (3) patients without adequate supplementary examinations for this study, including DHI, caloric tests, vHITs and MRI. In total, 58 patients met the inclusion criteria for this study. Patients with more than 3 remissions per day, each lasting over 1 h were assigned to the episodic vertigo (EV) group, and subjects without significant relief into the sustained vertigo (SV) group. The flow chart for screening and grouping patients is shown in Figure 1.

### Vestibular function test

Vestibular function tests, composed of nystagmus evaluation, bilateral caloric tests and vHITs, were performed within 2 days after the first visit. All patients were asked to take the tests during the symptomatic phases. An infrared-illuminated, vision-denied video nystagmography (VNG) goggle (VG40, ICS Medical Schaumburg, IL, USA) was used to record the intensity and direction of the slow-phase velocity (SPV) of SN. The SN was captured in darkness in the sitting position without visual fixation, when eyes are pointing straight ahead and laterally to either side. SPV toward the left was defined as positive, and toward right as negative.

Before a caloric test, an otoscope was used in excluding contraindications, such as tympanic membrane perforation. Patients were asked to place their heads on the pillows raised by 30°. Each ear was irrigated with constant airflow at alternating temperatures of 24 °C and 50 °C for 30 s (23). The nystagmus was recorded using an infrared video-based system (CHARTR VNG, ICS Medical Schaumburg, IL, USA). The maximum SPV of the nystagmus was calculated after each irrigation, and Jongkees' formula was used to determine CP.

The high-frequency VOR functions of horizontal and vertical canals were assessed by vHITs, which were applied in all the subjects using ICS impulse system (Otometrics, Denmark). The impulse system includes a pair of goggles with integrated video oculography camera and a half-silvered mirror. The subject's right eye was illuminated by a low-power infrared light-emitting diode, and was reflected into the camera by the mirror. The parameters of eyeball movements were thus recorded. A small sensor placed on the goggles was used to measure head movements of the subjects.

All examinations were performed by a skilled physician who specialized in neurotological testing and vHITs. The patients were asked to gaze at an earth-paralleled target 1.2 m in front during the examination. Firstly, calibration was performed by subjects staring at the left and right laser targets alternately. The horizontal canals were subsequently evaluated after excluding the interference of blinking or eyeballs-swaying. The physician stood behind a patient and turned the patient's head to the left and right unpredictably at a small angle (10°-20°) with an appropriate velocity (150°-200° per second). The movement of the head in the horizontal plane stimulated the lateral semicircular canals, and the movement was only in the atlanto-occipital joint pivoting around the odontoid apophysis axis (35). Next, the physician rotated the subject's head at a 45° angle relative to the trunk to evaluate the vertical semicircular canals. The head impulses were performed downward to stimulate the anterior canal opposite to the side of rotation, and upward to stimulate the posterior canal at the side of rotation (15). In a full test, 20 impulses were applied to each direction (36).

### Statistics

The threshold of statistical significance for differences was set *P* < 0.05 for each test. The Wilcoxon rank sum test was done to analyze ordered categorical data. As for quantitative data, the description was expressed as mean ± standard deviation (x ± s). Student's *t*-test was used to compare the quantitative data when they accorded with a normal distribution and their variance was homogeneous. Otherwise, the Wilcoxon signed rank sum test was used. The rates of the two groups were compared *via* Chi-square test. Correlation analysis was conducted to determine the relevance between variables of overt saccades, VOR gain and DHI score. Statistical analyses were performed using SPSS version 25.0 for Windows (SPSS Inc., Chicago, IL, USA).

## Results

**Figure 2:**
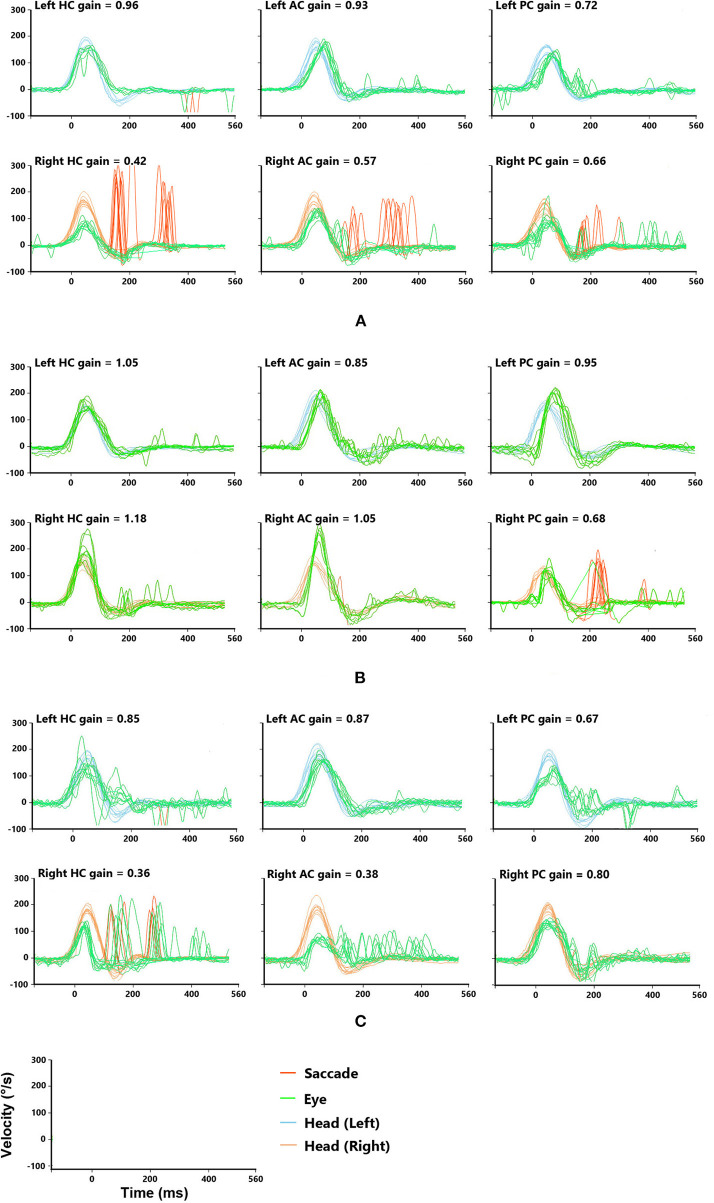
Typical vHITs images recorded from patients with episodic vertigo. The sample coordinates (last row) indicate the units of vHITs. **(A)** A patient with damaged superior and inferior vestibular nerves. The vHITs findings showed decreased VOR gains and corrective saccade for the right horizontal semicircular canal (HC) and vertical semicircular canals, including anterior and posterior semicircular canal (AC and PC). **(B)** A patient with damaged inferior vestibular nerve. The vHITs findings showed decreased gains in VOR for the right PC, while that for right the AC and HC was normal. **(C)** A patient with damaged superior vestibular nerve. The vHITs findings showed decreased gains of VOR for the right AC and HC. The VOR for right PC was normal.

We finally enrolled a total of 58 patients who experienced visual rotation or gait instability diagnosed with VN. The patients were composed of 29 males and 29 females, aged 21–83. More than half of them (31 subjects) went to a vertigo clinic within 5 days. The EV group composed of 11 subjects, whose average age was 58.27 ± 15.05, the SV group included 47 subjects, whose average age was 51.04 ± 15.21. In the EV group, 9 patients only had episodic vertigo from the onset to their initial consultation, whereas 2 patients had transient vertigo before the onset of prolonged symptoms. The demographic, and clinical characteristics of the subjects are shown in Tables 1, 2. For the typical vHIT results of the subjects, see Figure 2.

**Table 1:** Demographic, clinical characteristics and vHIT results of the 58 VN patients.

**No**.	**Gender**	**Age**	**Global duration of symptoms (days)**	**Duration** **of vertigo episodes (hours/** **day)**	**Frequency** **of vertigo episodes (daily)**	**DHI score**	**SN**	**SPV**	**Lesion**	**Ipsilesional** **VOR**			**Contralesional** **VOR**			**Mean latency of OS**	**Mean velocity of OS**	**CS**
										**LC**	**AC**	**PC**	**LC**	**AC**	**PC**			
1	F	33	14	>5	3–5	56	N	/	R	0.68	0.72	0.60	1.05	0.91	0.73	159.0	149.0	P
2	M	47	3	1–2	2; persistent symptoms set in after 1 day	38	P	1.8	R	0.42	0.57	0.66	0.96	0.93	0.72	283.0	135.7	N
3	F	50	7	> 5	1–2	34	P	−1	L	0.29	0.79	0.94	1.13	0.90	1.12	241.5	244.0	N
4	M	51	10	>5	3–5	72	N	/	L	0.37	0.56	1.00	1.20	0.98	1.18	213.0	261.0	N
5	M	51	24	2–5	2; persistent symptoms set in after 2 days	68	N	/	R	0.53	0.55	0.43	0.85	0.87	0.67	169.0	284.0	P
6	M	54	15	2–5	2–3	14	N	/	R	0.76	0.61	0.85	1.20	0.83	0.83	241.3	180.0	N
7	F	56	7	>5	1–2	60	N	/	L	0.41	0.44	0.99	1.20	0.78	1.07	152.0	162.0	P
8	M	63	7	> 5	3–4	12	N	/	R	0.69	0.54	0.64	1.12	0.75	0.68	292.0	297.0	P
9	M	75	2	>5	2–3	56	P	4	R	0.63	0.62	0.50	0.72	0.89	0.47	248.0	158.0	N
10	F	78	30	1–2	> 5	38	N	/	R	0.35	0.64	0.45	0.88	0.82	0.74	240.0	287.5	N
11	M	83	3	1–2	3–4	28	N	/	L	0.78	0.78	0.59	1.20	1.03	0.51	209.5	226.0	P
12	F	52	2.5	/	/	58	N	/	R	0.72	0.74	0.82	1.20	1.04	0.80	195.5	159.5	P
13	F	53	7	/	/	62	P	−5	L	0.06	0.38	0.24	0.72	0.95	0.44	120.0	118.7	N
14	F	54	3	/	/	68	P	1.2	R	0.60	0.45	0.91	0.88	0.77	0.72	297.0	105.5	N
15	M	32	10	/	/	30	P	−2	L	0.23	0.34	0.48	0.94	0.89	0.70	193.0	192.0	P
16	F	34	2	/	/	64	N	/	R	0.11	0.77	0.74	0.92	0.85	0.82	275.5	272.0	P
17	F	34	10	/	/	60	P	3.2	R	0.43	0.63	0.72	0.89	0.60	0.81	219.5	192.5	N
18	F	38	2	/	/	68	P	1	R	0.40	0.77	1.15	1.02	0.58	0.87	183.0	143.0	N
19	F	42	2	/	/	52	P	3.6	R	0.38	0.31	0.59	1.03	0.93	0.86	247.5	193.0	N
20	F	43	20	/	/	74	P	−4.8	L	0.56	0.43	0.82	0.60	0.91	0.82	258.0	224.0	N
21	M	45	10	/	/	56	N	/	R	1.20	0.87	0.56	1.20	0.85	0.95	286.5	172.0	P
22	M	48	5	/	/	50	P	4	R	0.79	0.48	0.89	1.20	1.00	0.85	254.5	178.5	N
23	M	49	2	/	/	68	P	−4.7	L	0.20	0.30	0.51	0.84	0.58	0.75	278.0	296.0	N
24	F	67	3	/	/	50	N	/	R	0.78	0.73	1.09	1.06	1.06	0.88	116.0	174.0	N
25	F	67	7	/	/	32	N	/	R	0.40	0.44	0.83	0.98	0.94	0.75	376.0	359.0	N
26	M	68	10	/	/	38	P	−3	L	0.77	0.93	0.47	1.12	0.94	0.71	213.0	160.0	N
27	M	73	14	/	/	20	N	/	L	0.65	0.66	0.44	0.87	0.92	0.68	306.0	136.5	N
28	F	59	30	/	/	76	N	/	R	1.07	0.87	0.68	1.11	1.09	0.77	190.0	229.0	N
29	M	75	2	/	/	66	P	5	R	0.77	0.75	0.91	0.81	0.95	0.79	310.0	140.0	N
30	F	76	7	/	/	60	N	/	R	0.52	0.22	0.74	0.97	0.58	0.42	208.0	200.0	N
31	F	83	2	/	/	46	N	/	L	0.78	0.66	0.69	1.19	0.83	0.72	234.0	84.5	N
32	M	64	2	/	/	68	P	−2.4	L	0.44	0.34	0.87	0.81	0.80	0.82	293.5	221.0	P
33	M	53	2.5	/	/	20	P	−3	L	0.25	0.53	0.87	0.82	0.87	0.72	288.5	182.5	N
34	F	67	10	/	/	76	N	/	R	0.74	0.78	0.70	1.04	0.81	0.82	248.5	159.0	N
35	F	61	3	/	/	62	P	3	R	0.50	0.64	0.33	0.75	0.65	0.78	216.0	163.5	P
36	M	30	24	/	/	62	N	/	R	0.67	0.78	0.95	0.88	0.96	1.20	276.0	168.0	P
37	M	61	3	/	/	60	P	2	R	0.36	0.38	0.42	0.85	0.87	0.67	171.0	130.0	N
38	F	57	2.5	/	/	74	P	5	R	0.38	0.43	0.73	1.05	0.89	0.83	338.0	197.0	N
39	M	37	1.2	/	/	68	P	2	R	0.72	0.57	1.05	1.20	1.05	1.13	265.0	274.0	P
40	M	62	3	/	/	30	P	−5	L	0.36	0.41	0.98	0.93	1.11	0.75	252.0	243.3	P
41	F	28	4	/	/	56	P	2	R	0.34	0.70	0.88	0.95	0.94	0.94	341.0	225.0	N
42	M	36	2.5	/	/	70	P	1	R	0.97	1.02	0.83	0.32	0.51	1.11	201.5	225.5	N
43	F	21	1.5	/	/	60	P	5	R	0.32	0.46	0.87	1.03	0.80	0.86	240.0	199.5	P
44	F	57	15	/	/	62	P	1	R	0.55	0.59	0.89	0.88	0.95	0.81	224.5	217.5	N
45	F	36	2	/	/	62	P	2	R	0.67	0.78	0.90	1.02	1.00	0.85	185.0	299.0	N
46	M	58	15	/	/	58	N	/	R	0.80	1.07	0.76	0.47	0.38	0.86	250.0	223.5	P
47	F	48	7	/	/	70	P	3	L	0.87	0.87	0.84	0.57	0.78	0.85	238.0	200.0	N
48	M	53	3	/	/	68	N	/	L	0.45	0.64	0.87	1.07	1.18	0.78	304.0	160.5	P
49	F	26	4	/	/	62	N	/	R	0.78	0.71	0.53	1.04	0.81	0.81	219.3	196.3	N
50	M	64	3	/	/	20	P	−6	L	0.26	0.49	0.51	1.00	0.69	0.63	283.5	168.5	P
51	M	38	2.5	/	/	70	P	−2.8	L	0.63	0.51	1.17	1.20	1.05	1.05	181.0	274.0	P
52	F	30	3	/	/	70	P	−1	L	0.48	0.53	0.78	0.86	1.07	0.70	242.5	170.5	P
53	M	48	32	/	/	76	N	/	L	0.31	0.67	0.65	0.99	1.02	0.81	228.7	147.7	P
54	F	58	22	/	/	38	N	/	R	1.00	0.94	0.92	0.59	0.55	0.49	245.7	158.0	P
55	M	45	14	/	/	60	P	1.4	R	0.48	0.36	0.78	1.09	0.87	0.87	299.3	220.7	P
56	M	60	2	/	/	68	P	−2	L	0.70	0.45	0.80	1.19	1.00	0.72	257.5	167.5	N
57	F	56	7	/	/	42	P	−2.4	L	0.46	0.48	0.56	1.18	0.97	0.73	253.0	178.0	P
58	M	63	1.5	/	/	72	P	−5	L	0.37	0.41	0.73	1.18	1.02	0.77	292.0	272.0	N

F, female; M, male; N, negative; P, positive; LC, Lateral semicircular canal; AC, Anterior semicircular canal; PC, posterior semicircular canal; SN, spontaneous nystagmus in the sitting position; SPV, SPV of SN while looking straight-ahead in the sitting position; OS, overt saccades; CS, covert saccades.

**Table 2:** Clinical characteristics of all patients with vestibular neuritis.

		***n* %**
Gender	Male	29 (50.0)
	Female	29 (50.0)
Age (years)	≤65	47 (81.0)
	>65	11 (19.0)
Course (days)	≤5	31 (53.4)
	>5	27 (46.6)
Episodic vertigo	Positive	11 (19.0)
	Negative	47 (81.0)
History of prodromal infection	Positive	13 (22.4)
	Negative	45 (77.6)
Vestibular symptoms	Dizziness	15 (25.9)
	Oscillopsia	44 (75.9)
	Instability	36 (62.1)
Autonomic symptoms	Nausea	50 (86.2)
	Vomiting	44 (75.9)
Spontaneous nystagmus	Positive	33 (56.9)
	Negative	25 (43.1)
Impaired vestibular nerve	Superior	34 (58.6)
	Inferior	2 (3.4)
	Both	22 (37.9)
Compensatory saccade	Covert	24 (41.4)
	Overt	58 (100)
DHI questionnaire score	Mild to moderate (≤60)	31 (53.4)
	Severe (> 60)	27 (46.6)

Patients were treated with 40 mg of pulse methylprednisolone, which was reduced by half every 3 days, as well as physical rehabilitation. Oral anxiolytics was initiated for some patients with severe vertigo after consulting the psychiatrist. All the patients were relieved of their symptoms after 2 weeks of treatment. In the first year of follow-up, no patient reported the recurrence of vertigo or gait instability.

**Figure 3:**
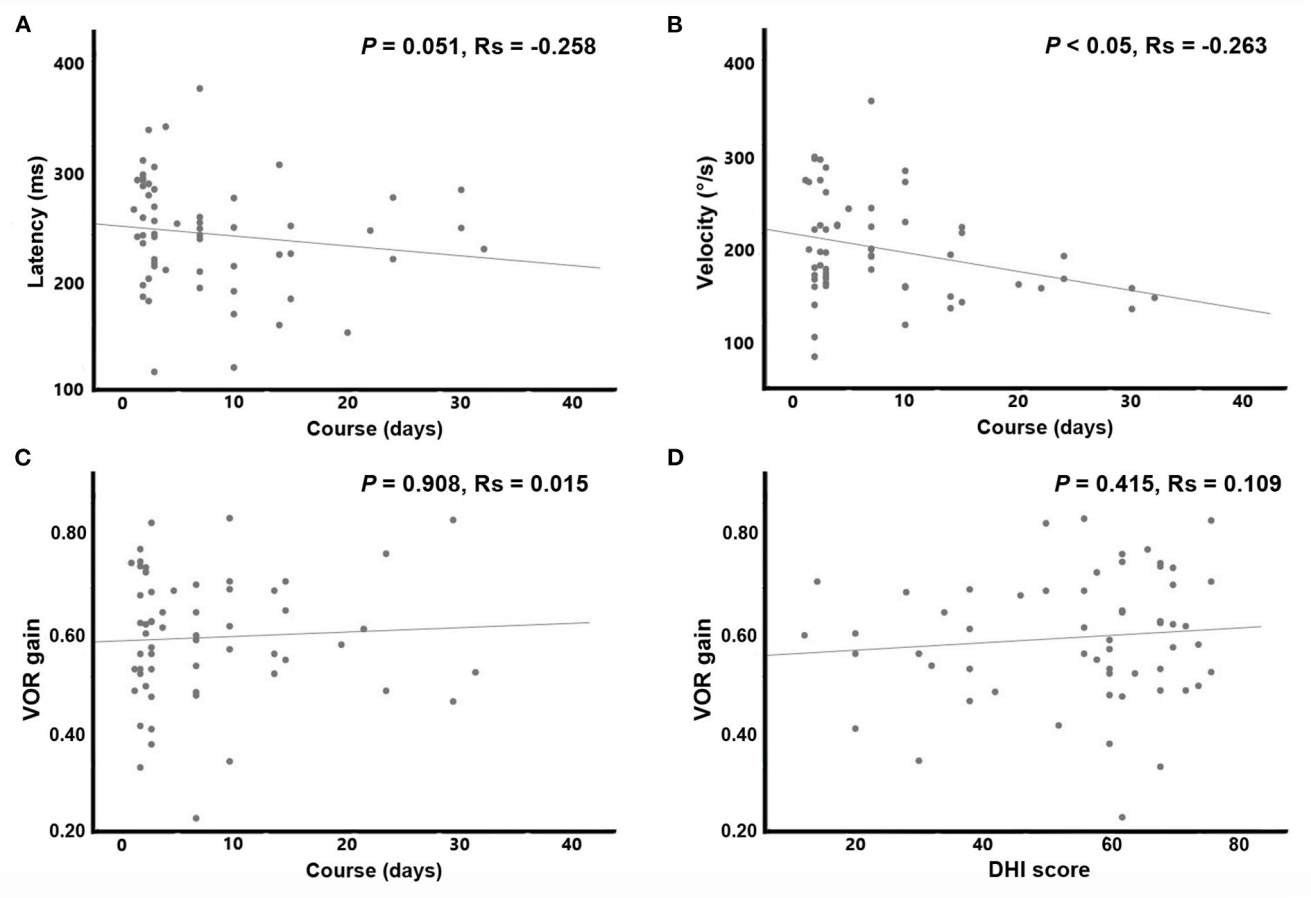
Scatterplots and results of Spearman correlation. With a longer disease course, the velocity of overt saccade was smaller (*p* < 0.05, Rs = −0.263) in patients with VN. **(A)** Abscissa: disease course (days); ordinate: latency of overt saccades (ms). **(B)** Abscissa: disease course (days); ordinate: velocity of overt saccades (°/s). **(C)** Abscissa: disease course (days); ordinate: VOR gain. **(D)** Abscissa: DHI score (points); ordinate: VOR gain.

Statistical analysis revealed that compared to the EV group, patients in the SV group had significantly higher occurrence of SN and proportion of severe vertigo (DHI questionnaire score > 60). No significant differences were found in gender, age, disease course, prodromal infection history, incidence of covert saccade, impaired vestibular nerve and bilateral VOR difference between the two groups. Since the data of disease course and DHI score accorded with skewed distribution, Spearman correlation analysis was applied to reveal the associations of the overt saccade (latency and velocity), VOR gain and disease course. Statistical analysis reveal that the velocity of overt saccade was negatively correlated with disease course (*P* < 0.05, Rs = −0.263) in patients with VN. Detailed statistical results are shown in Table 3 and Figure 3.

**Table 3:** Statistical analysis between patients in the episodic vertigo (EV) group and the sustained vertigo (SV) group.

		**EV**	**SV**	**t /z/x^2^ value**	***P* value**	**OR (95% CI)**
Age (years)		58.27 ± 15.05	51.04 ± 15.21	−1.42	0.16	-
Lantency of overt saccade (ms)		234.44 ± 42.88	244.39 ± 53.41	0.58	0.57	-
Velocity of overt saccade (°/s)		186.18 ± 38.82	203.66 ± 57.84	0.95	0.35	-
Disease course (days)		7.00 (3.00, 15.00)	3.50 (2.00, 10.00)	−1.76	0.08	-
Bilateral VOR difference		0.29 ± 0.10	0.28 ± 0.12	−0.24	0.81	-
History of prodromal infection	Positive	3 (27.3 %)	10 (21.3 %)	0.001	0.98	-
	Negative^*^	8 (72.7 %)	37 (78.7 %)			
Spontaneous nystagmus in straight-ahead position	Positive	2 (18.2 %)	31 (66.0 %)	6.46	***0.01***	8.72 (1.68–45.25)
	Negative^*^	9 (81.8 %)	16 (34.0 %)			
Covert saccades	Positive	4 (36.4 %)	20 (42.6 %)	0.001	0.97	-
	Negative^*^	7 (63.6 %)	27 (57.4 %)			
Impaired vestibular nerve	Superior or inferior^*^	6 (54.5 %)	30 (63.8 %)	0.54	0.83	-
	Both	5 (45.5 %)	17 (36.2 %)			
Gender	male^*^	7 (63.6 %)	22 (46.8 %)	1.01	0.32	-
	female	4 (36.4 %)	25 (53.2 %)			
DHI questionnaire score	Mild to moderate (≤60)^*^	9 (81.8 %)	22 (46.8 %)	4.39	***0.04***	5.11 (1.00–26.25)
	Severe (> 60)	2 (18.2 %)	25 (53.2 %)			

* The control group in chi-square test. Statistical analysis revealed that patients with spontaneous nystagmus had significantly higher occurrence of sustained vertigo (*P* < 0.05, OR = 8.72). Besides, patients with severe vertigo (DHI questionnaire score > 60 points) had significantly higher occurrence of sustained vertigo (*P* < 0.05, OR = 5.11). *P*-value less than 0.05 are exhibited in bold and italic font.

## Discussion

The typical manifestations of VN include severe prolonged vertigo and SN. With the advancement in vestibular function testing which evaluate different portions of the peripheral vestibular system, the diagnosis of VN is no longer restricted to symptomatology and limited supplementary tests. For example, vHITs permit evaluation of the angular VOR in the plane of all six semicircular canals (15, 28). Other quantitative assessments also provide more indicators other than CP, such as VOR gain, saccades, VEMP response and deflection angle of SVV, to assess the clinical course and prognosis of VN (24, 26, 37). Benefited from advances in vestibular testing, an increasing number of episodic atypical syndromes in VN were therefore recognized. According to Lee (28), approximately a quarter of patients experienced transient dizziness before severe prolonged vertigo. Silvoniemi (3) found that 8.6% of patients with VN had a mild prodromal sensation of unsteadiness 1–7 days before the onset of intensive vertigo. To the best of our knowledge, no study has systematically analyzed the characteristics of VN patients with episodic vertigo. This study presented those 11 (19.0%) patients with VN and episodic sense of vertigo, among whom 2 (3.4 %) had episodic manifestation before the prolonged syndrome onset. Since this study aimed to disclose the episodic syndromes in patients with VN, we assigned the 2 cases into the EV subgroup according to the grouping criteria.

There were 31 patients (53.4 %) enrolled in this study within 5 days from the onset. 12 of whom were re-examined for the vHITs at discharge. However, we did not track their vHITs in the early stages in this study. By sequentially measuring the VOR gains of patients with acute VN, previous studies have found the ipsilesional VOR gains vary after the initial measurement during the week from onset (38, 39). Meanwhile, patients with lower initial VOR gains were less likely to improve on subsequent 3–5 days, and would generate more covert catch-up saccades over time (39).

Compared to those with sustained vertigo, patients with episodic syndrome reported lower incidence of SN. In this study, SN referred to the nystagmus recorded while patients were looking in the straight-ahead (center gaze) position (40). Patients enrolled in this study without SN presented with first degree vestibular nystagmus while gazing at the contralesional side, indicating an acute phase of VN along with other inclusion criteria. According to previous researches, the intensity of SN could be modified in one or more other gaze positions (40). Since SN is the most prominent indicator of static vestibular imbalance, which could determine the severity of clinical symptoms (40, 41), a lower incidence of SN implies a milder lesion of static imbalance and/or an improvement of nerve function after initial loss in patients with episodic syndrome (41). We thus presumed that patients in the EV group either had milder VOR deficiency or abided by more effective strategies of central compensation than those in the SV group.

To assess the behavioral and emotional status, patients were required to complete the DHI questionnaire at initial consultation. In this study, patients in the SV group had significantly higher occurrence of severe vertigo handicap (DHI score > 60) than those in the EV group. In other words, the life quality of patients in the SV group was considerably more likely to be affected by physical disability and psychological stress. This is in line with previous studies that patients with vestibular hypofunctional exhibited increasing incidence of anxiety and depressions (42, 43). In our research, a portion of patients with severe vertigo and emotional dysfunction were administered with oral anxiolytics after consulting the psychiatrist. At the 1-year follow-up, these patients presented no emotional disorders. From a therapeutic point of view, timely psychological treatment or drug intervention should be considered for patients with high DHI score to prevent emotional disorders.

In our research, two cases had inferior vestibular nerve affected only, and both reported sustained vertigo. The small sample size of inferior neuritis showed limited impact on statistical results. We analyzed the incidence of incomplete vestibular nerve (superior or inferior) impairment between the two groups, and no statistical differences were identified.

Of note, our study revealed that neither the VOR asymmetry between the subgroups showed a difference, nor did the VOR gain correlate with the DHI score in overall patients. Although the quantitative lesion indicators are commonly irrelevant to the intensity of symptoms in clinical practice, negative results without correlations are rarely seen in literature. Limited cases reported that some patients had physical signs of VN, but their ipsilesional VOR gains were in the normal range (39). By sequential head impulse measurements, Palla et al. found that VOR gains toward the ipsilesional side appeared to descend initially and then increased with the course, which was not parallel to the disease progression (38). From another perspective, the DHI questionnaire comprised 25 items with scores ranging from 0 and 100 points. It could be further subdivided into physical, functional, and emotional scores (44). The ipsilesional VOR gains could reflex the intensity of physical symptoms, but not the emotional and functional disabilities. Previous studies have shown that anxiety and dizziness are co-morbid symptoms in a larger percentage of patients (42). Emotion disorders could aggravate clinical symptoms (45–47) and thus impact the DHI score. Further study with multivariate analysis and larger sample size would be helpful to reveal the relationship between symptoms and lesion indicators.

In this study, 24 patients presented with both overt and covert saccades at vHITs, while 34 patients had overt saccades only. The velocity of overt saccades presented a negative correlation with the disease course in overall VN patients. This is in line with a previous study that the velocity of covert and overt saccade could exhibit a gradual decrease during follow-ups, while the latency of them remained unchanged (37). Triggered by the sensory stimulus from the cervico-ocular reflex (COR), covert saccades are considered facilitating dynamic compensation to overcome the inadequate VOR on the ipsilesional side (31, 38, 48). Overt saccades, as secondary catch-up saccades, are generated when the covert saccades fail to drive eye-movements reaching the target during head rotation. In line with previous study, our study found a certain number of patients presenting with covert saccades at early stage of VN (39). Therefore, the incidence and parameters of saccades, covert or overt, are a promising indicator for evaluation of vestibular deficit and central compensation other than VOR (49). We postulated that there could be a different saccadic strategy between the EV and the SV groups, which might facilitate vestibular recovery and impact the manifestations. However, neither the incidence of overt saccades nor the parameters of covert saccades showed a significant difference between the two subgroups yet. Further investigation of larger samples might provide a better understanding of the different patterns of saccades in VN patients with episodic vertigo.

To date, diagnosis of VN remains to be exclusively based on symptomatology, and no supplementary testing can be used as a gold standard. Therefore, even with varied auxiliary results for reference, we still could not totally rule out the possibilities of alternative episodic vestibular syndromes in the enrolled patients with EV, such as a first manifestation of vestibular migraine or Meniere disease, not even a mild form of ischemic disease. Some supporting foundation would be that all patients exhibited a normal cranial MRI, and none of them had another episode during the one-year follow-up. Meanwhile, to make the VN diagnosis as accurate as possible, patients with auditory symptoms or a history of diabetes, migraine or anxiety were also excluded in this study. However, a long term follow-up is still warranted to fully exclude the possibility of other diseases.

This study has a few limitations. Firstly, the interference of personal emotions with the results was unavoidable. For example, in patients with severe vertigo and higher DHI score, the duration of remission period may have been subjectively shortened. To avoid the statistical bias, we used standard tabulated questionnaires, checking with families and telephone follow-ups to mutually verify the reliability of symptomatology data. Secondly, this study contained many variables whereas the sample size was only 58, a much smaller proportion of which exhibit of episodic vertigo. It is inappropriate to operate Logistic regression fitting the factors contributing to episodic vertigo, thus making the findings of this study unstable. Further prospective studies with a larger sample size would be established to vertify the value of parameters of SN and DHI score in VN diagnosis with episodic vertigo.

## Conclusion

Our findings support the following conclusion: (1) some patients with VN may have episodic vertigo and instability; (2) non-sustained manifestations in VN patients overlap with other vestibular disorders and stroke-related vertigo, which add an additional layer of complexity to the differential diagnosis of new onset episodic vertigo; (3) our retrospective analysis of the clinical characteristics and vHIT parameters reveals the atypical features and potential pathophysiological mechanism of episodic syndromes in VN; and (4) VOR gain and saccades measured by vHIT promise to be reliable indicators for vestibular rehabilitation process.

## Data availability statement

The original contributions presented in the study are included in the article/supplementary material, further inquiries can be directed to the corresponding authors.

## Ethics statement

The studies involving human participants were reviewed and approved by the Ethics Committee of the Affiliated Brain Hospital of Nanjing Medical University. Written informed consent to participate in this study was provided by the participants' legal guardian/next of kin.

## Author contributions

LT conceived the content, wrote the preliminary version of the manuscript, and interpreted the data. WJ contributed to the patients' follow-ups, conceptualization of the content, and co-writing the academic content related to neurology. XW revised the article and approved the final manuscript before submission. All authors contributed to the article and approved the submitted version.

## Conflict of interest

The authors declare that the research was conducted in the absence of any commercial or financial relationships that could be construed as a potential conflict of interest.

## Publisher's note

All claims expressed in this article are solely those of the authors and do not necessarily represent those of their affiliated organizations, or those of the publisher, the editors and the reviewers. Any product that may be evaluated in this article, or claim that may be made by its manufacturer, is not guaranteed or endorsed by the publisher.
